# 4293 Relationship between recent drinking history, subjective response to alcohol, and sex in HRV in non-dependent drinkers

**DOI:** 10.1017/cts.2020.143

**Published:** 2020-07-29

**Authors:** Sachin Sundar, Bethany L. Stangl, Reza Momenan, Vijay A. Ramchandani, Kristin Corey, Khem Plata

**Affiliations:** 1 National Institutes of Health

## Abstract

OBJECTIVES/GOALS: Previous research has shown acute and chronic alcohol effects on cardiac function, including elevated heart rate (HR) and lowered heart rate variability (HRV). This study aimed to examine the relationship between cardiac reactivity and subjective response following intravenous (IV) alcohol in non-dependent drinkers. METHODS/STUDY POPULATION: Non-dependent drinkers (N = 46, average age = 25.2) completed a human laboratory IV alcohol self-administration (IV-ASA) session. Subjective response to alcohol was assessed using the Drug Effects Questionnaire (DEQ) and Alcohol Urge Questionnaire (AUQ). Drinking behavior was assessed using the Alcohol Timeline Followback (TLFB) and Alcohol Use Disorders Identification Test (AUDIT). HR was recorded using the Polar Pro Heart Rate monitor throughout the session. HRV measures were calculated using guidelines determined by the Task Force of the European Society of Cardiology and The North American Society of Pacing and Electrophysiology. RESULTS/ANTICIPATED RESULTS: Recent drinking history as measured by the AUDIT and TLFB was not significantly different by sex. Results showed heavier drinking measures (AUDIT and TLFB) were positively associated with HRV measures (all p-values < 0.02). Those who reported a greater increase in alcohol craving (AUQ score) and wanted more alcohol (DEQ) following an alcohol prime, showed a greater change in HRV (p < 0.005). When examining HRV change from baseline throughout the priming session, there was a significant sex interaction for NN50 (p < 0.03) and a trend for PNN50 (p-value < 0.07). DISCUSSION/SIGNIFICANCE OF IMPACT: Acute IV alcohol alters cardiac reactivity measures in non-dependent drinkers. Future directions include examining the role of sex in HRV changes during alcohol consumption during IV-ASA. Understanding the effect of alcohol on cardiac reactivity and physiology may help characterize those at risk for alcohol use disorders.

